# Supplementary material for: Observation of Magnetic‐Anisotropy Crossover and High‐Temperature Skyrmions in the Dirac Magnet Fe3Ge with a Distorted Kagome Lattice
Source: Adv Sci (Weinh). 2026 Aug 24;13(63):e77344. doi: 10.1002/advs.77344 (PMC13502365; doi:10.1002/advs.77344)
Supplement: Supplementary file 1 [file ADVS-13-e77344-s001.docx]

**Supporting Information for** **Observation of Magnetic-Anisotropy Crossover and High-Temperature Skyrmions in the Dirac Magnet Fe_3_Ge with a Distorted Kagome Lattice**

Yalei Huang^1,12,^ Xiaowei Lv^2,12^, Bin Li^3^, Chunqiang Xu^4, *^, Dhanarajagopal Alltrin^5^, Xiaoxuan Ma^6^, Wanting Yang^6^, Wei Zhou^7, *^, Xiangzhuo Xing^8^, Wen-Chin Lin^9^, Raman Sankar^5^, Michael Smidman^10^, Shixun Cao^6^, Dong Qian^11^, Renchao Che^2, *^ and Xiaofeng Xu^1, *^

^1^Zhejiang Provincial Key Laboratory of Quantum Precision Measurement, School of Physics and Optical Engineering, Zhejiang University of Technology, Hangzhou 310023, China

^2^Laboratory of Advanced Materials, Shanghai Key Lab of Molecular Catalysis and Innovative Materials, State Key Laboratory of Coatings for Advanced Equipment, College of Smart Materials and Future Energy, Fudan University, Shanghai 200438, China

^3^School of science, Nanjing University of Posts and Telecommunications, Nanjing 210023, China

^4^School of Physical Science and Technology, Ningbo University, Ningbo 315211, China

^5^Institute of Physics, Academia Sinica, Nankang, Taipei, R.O.C. 11529 Taiwan

^6^Materials Genome Institute, Shanghai University, Shanghai 200444, China

^7^School of Electronic and Information Engineering, Suzhou University of Technology, Changshu 215500, China

^8^Laboratory of High Pressure Physics and Material Science (HPPMS), School of Physics and Physical Engineering, Qufu Normal University, Qufu 273165, China

^9^Department of Physics, National Taiwan Normal University, Taipei 116, Taiwan

^10^School of Physics, Zhejiang University, Hangzhou, 310058, ‌China

^11^Key Laboratory of Artificial Structures and Quantum Control (Ministry of Education), School of Physics and Astronomy, Shanghai Jiao Tong University, Shanghai 200240, China

^12^These authors contributed equally: Yalei Huang, Xiaowei Lv.

^*^Corresponding authors: Chunqiang Xu, Wei Zhou, Renchao Che, Xiaofeng Xu.

**Email:** xuchunqiang@nbu.edu.cn, wei.zhou@cslg.edu.cn, rcche@fudan.edu.cn, xuxiaofeng@zjut.edu.cn

**This file includes:**

**1. Single-crystal X-ray** **characterization of Fe_3_Ge**

**2. Large intrinsic anomalous Hall effect and topological Hall effect in Fe_3_Ge**

**3.** **Anisotropic magnetoresistance effect in Fe_3_Ge**

**4. Band structure of Fe_3_Ge in three distinct ferromagnetic phases**

**5. Easy-plane and easy-axis magnetic anisotropy in Fe_3_Ge**

**6. Lorentz transmission electron microscopy in Fe_3_Ge**

**7. Mechanisms of skyrmion formation**

**8. Comparison of centrosymmetric ferromagnets**

**9. Magneto-optical Kerr effect measurement setup**

**10. Geometric tunability of mixed textures in Fe_3_Ge**

**11. Video of skyrmion formation**

# 1. Single-crystal X-ray characterization of Fe_3_Ge

To examine the crystalline structure of Fe_3_Ge single crystals, the crystal was mounted on a nylon loop using Paratone oil and analyzed using a Rigaku XtalLAB Synergy, Dualflex, Hypix single-crystal X-ray diffractometer at room temperature, as shown in Fig. S1.

**Figure S1.** Fe_3_Ge single-crystal X-ray diffraction pattern.

The results of the single-crystal X-ray diffraction analysis are summarized in Tables S1 and S2. The crystal structure was solved in the space group *P*6_3_/*mmc*. The formula unit, Fe_3_Ge, corresponds to *Z* = 2. The final anisotropic full-matrix least-squares refinement on F^2^, with 8 variables, converged to *R*_1_ = 6.52% for observed data and *wR*_2_ = 14.50% for all data, with a goodness-of-fit of 1.066. The final difference electron density synthesis revealed a maximum peak of 4.58 e^-^/Å^3^ and a minimum hole of -5.52 e^-^/Å^3^, with an RMS deviation of 1.200 e^-^/Å^3^. Based on the refined model, the calculated density was 8.108 g/cm³, and the F(000) value was 220.0 e^-^. The SCXRD analysis confirms the high quality of the grown crystals.

**Tables S1.** The crystal structure and refinement of Fe_3_Ge at room temperature

| Chemical Formula Fe_3_Ge | |
| --- | --- |
| Formula Weight | 240.14 g/mol |
| Space Group | *P*6_3_/*mmc* |
| *a* | 5.1814(6) Å |
| *b* | 5.1814(6) Å |
| *c* | 4.2308(5) Å |
| *V* | 98.37(3) Å^3^ |
| Z | 2 |
| Density | 8.108 g/cm^3^ |
| Absorption coefficient | 36.293 mm^−1^ |
| F (000) | 220.0 e^-^ |
| 2*θ* range | 9.084° - 81.182° |
| Reflections collected | 773 |
| Independent reflections | 145 [*R*_int_ = 0.0709] |
| Refinement method | Full-matrix least-squares on F^2^ |
| Data/restraints/parameters | 145/0/8 |
| *R*_1_ (I>2σ(I)) | 0.0608 |
| *wR*_2_ (I > 2 σ(I)) | 0.1381 |
| *R*_1_ (all) | 0.0652 |
| *wR*_2_ (all) | 0.1450 |
| Largest diff. peak and hole | +4.58 e^-^/Å^3^ and -5.52 e^-^/Å^3^ |
| R. M. S. deviation from mean | 1.200 e^-^/Å^3^ |
| Goodness-of-fit on F^2^ | 1.066 |

**Table S2.** Atomic coordinates and equivalent isotropic atomic displacement parameters (Å^2^) of Fe3Ge. (*U_eq_* is defined as one-third of the trace of the orthogonalized *U_ij_* tensor.)

|  | **Fe** | **Ge** |
| --- | --- | --- |
| **Wyck.** | 6*h* | 2*d* |
| ***x*** | 0.16347(7) | 1/3 |
| ***y*** | 0.32694(7) | 2/3 |
| ***z*** | 1/4 | 3/4 |
| **Occ.** | 1 | 1 |
| ***U_eq_*** | 0.002(4) | 0.001(4) |

# 2. Large intrinsic anomalous Hall effect and topological Hall effect in Fe_3_Ge

To understand the nature of anomalous Hall effect (AHE) in Fe_3_Ge, we calculated the Hall conductivity $\sigma_{xz}$. Considering the anisotropic nature of longitudinal resistivity (Fig. S2a), the $\sigma_{xz}$ was estimated using the relation,

$$\sigma_{xz}= {-\rho_{xz}}/{(\rho_{xx}\rho_{zz}+\rho_{xz}^{2})}$$

Fig. S2b displays the magnetic field dependence of $\sigma_{xz}$ at various temperatures. The value of anomalous Hall conductivity ($\sigma_{xz}^{A}$) can be acquired by extrapolating of high-field $\sigma_{xz}$ data to the zero field. The temperature dependent $\sigma_{xz}^{A}$ is presented in Fig. S2c. The $\sigma_{xz}^{A}$ initially increases with temperature, reaches a maximum of $\sim390 \Omega^{-1}\mathrm{cm}^{-1}$at 250 K, and subsequently decreases upon further warming. To further clarify the AHE origin, we analyzed the scaling of $\rho_{xz}^{A}$ vs $\rho_{xx}\rho_{zz}$, where $\rho_{xz}^{A}$ was obtained as the intercept of a linear fit to $\rho_{xz}$vs $\mu_{0}H$ at the high field region, as demonstrated in Fig. S2d. As shown in Fig. S2e, $\rho_{xz}^{A}$ exhibits an almost linear dependence on $\rho_{xx}\rho_{zz}$, implying that the AHE is governed predominantly by the intrinsic mechanism or extrinsic side-jump scattering. Theoretically, the side-jump contribution is expected to be much smaller, on the order of (${E_{so}}/{E_{F}}){e^{2}}/{ha_{z}}$, where *e* is electronic charge, *h* is the Plank constant, $a_{z}$ is the lattice parameter, $E_{so}$ is the spin-orbit coupling energy*,* and $E_{F}$ is the Fermi energy. Considering ${E_{so}}/{E_{F}}\approx{10}^{-3}-{10}^{-1}$, the estimated side-jump term is significantly lower than the experimentally observed $\sigma_{xz}^{A}$. This strongly suggests that the AHE in Fe_3_Ge is dominated by the intrinsic mechanism, which is consistent with prior reports ^1-3^.

**Figure S2.** **a** Temperature dependence of longitudinal resistivity $\rho_{xx}$ (*I* ∥ *x*) and $\rho_{zz}$ (*I* ∥ *z*), respectively. Note that [2$\bar{1}\bar{1}0$], [0$1\bar{1}0$] and [0001] directions are respectively denoted to be *x*, *y* and *z*. **b** Magnetic field dependence of Hall conductivity $\sigma_{xz}$ at various temperatures. **c** Temperature dependence of experimental anomalous Hall conductivity $\sigma_{xz}^{A}$. **d** Magnetic field dependence of Hall resistivity $\rho_{xz}$ at 300 K. The red solid line is the linear fitting. **e** Plot of $\rho_{xz}^{A}$ vs $\rho_{xx}\rho_{zz}$. The red solid line is the linear fitting. **f** Theoretically calculated energy dependence of intrinsic anomalous Hall conductivity $\sigma_{xz}^{A}$ with the magnetic moment along the in-plane lattice vectors.

Moreover, the slope of linear fit to $\rho_{xz}$vs $\mu_{0}H$ yields an intrinsic $\sigma_{xz}^{A}=392\Omega^{-1}\mathrm{cm}^{-1}$*.* Considering the integration of Berry curvature over the occupied states, we theoretically calculated the Fermi-level-dependent $\sigma_{xz}^{A}$ of Fe_3_Ge, as shown in Fig. S2f. In Fe_3_Ge, since the topological band states are concentrated near the $E_{F}$, a slight shift in $E_{F}$ will significantly influence the $\sigma_{xz}^{A}$, leading to a sharp peak near $E_{F}$. A large intrinsic $\sigma_{xz}^{A}$ of about $420\Omega^{-1}\mathrm{cm}^{-1}$ is observed near the $E_{F}$, which is in good agreement with the experiments. These findings indicate that the topological band structures play a crucial role in enhancing the intrinsic AHE in Fe_3_Ge.

**Figure S3.** Procedure implemented for obtaining the topological Hall resistivity $\rho_{yx}^{T}$. **a** Magnetic field dependence of $\rho_{xz}$ and the magnetization measured at 300 K with *I* ∥ *z*, *μ*_0_*H* ∥ *y*. **b** Plot of $\rho_{xz}/\mu_{0}H$ versus $M/\mu_{0}H$under the same measurement conditions at 300 K, where the red line indicates a linear fit. **c** Magnetic field dependence of the Hall resistivity $\rho_{xz}$, the calculated normal and anomalous Hall resistivity${R_{0}\mu}_{0}H +{R_{s}M}$, and the topological Hall resistivity $\rho_{yx}^{T}$. **d** Magnetic field dependence of topological Hall $\rho_{xz}^{T}$ at some selected temperatures. **e** Magnetic field dependence of magnetization and topological Hall resistivity at 300 K.

By including topological Hall effect (THE), the total Hall contribution can be expressed as,

$$\rho_{yx}=\rho_{yx}^{N}+\rho_{yx}^{A}+\rho_{yx}^{T}=R_{0}\mu_{0}H+R_{s}M+\rho_{yx}^{T}$$

Fig. S3 shows the procedure used for obtaining $\rho_{yx}^{T}$. The $\rho_{yx}^{T}$ vanishes when $\mu_{0}H>{\mu_{0}H}_{s}$ due to the consistent arrangement of magnetic moments and thus no spin chirality. Therefore, for $\mu_{0}H>{\mu_{0}H}_{s}$, the total Hall contribution can be expressed as:

$$\rho_{yx}=\rho_{yx}^{N}+\rho_{yx}^{A}=R_{0}\mu_{0}H+R_{s}M$$

which can be rewritten as:

$$\rho_{yx}/\mu_{0}H=R_{0}+R_{s}M/\mu_{0}H$$

As shown in Fig. S3b, a linear fit of the $\rho_{yx}/\mu_{0}H$ versus $M/\mu_{0}H$ curve at high magnetic field ($\mu_{0}H>{\mu_{0}H}_{s}$) yields the intercept $R_{0}$and the slope $R_{s}$. Subsequently, the contributions of the normal Hall effect ($\rho_{yx}^{N}=R_{0}\mu_{0}H$) and anomalous Hall effect ($\rho_{yx}^{A}=R_{s}M$) can be calculated. Finally, as shown in Fig. S3c, the $\rho_{yx}^{T}$ is determined by subtracting the calculated contributions ($\rho_{yx}^{N}+\rho_{yx}^{A}$) from the measured Hall data. In our present samples, we observe a clear THE signal after subtracting the normal Hall effect and the anomalous Hall effect, as shown in Fig. S3d. The magnitude and temperature dependence of the THE are comparable to prior reports ^2,4^.

As shown in Fig. S3e, this contribution peaks at approximately 0.6 T at 300 K, where the magnetization reaches about 90% of its saturation value rather than full saturation, indicating the presence of residual non-collinear spin components. For reference, a canting angle of approximately 26° corresponds to a 90% net moment projection (cos 26° ≈ 0.90). As discussed in the main text, Fe_3_Ge exhibits spin reorientation from the out-of-plane alignment above *T*_SR_ to the in-plane alignment at lower temperatures. Zero-field neutron diffraction confirms the coexistence of in-plane and out-of-plane magnetic moments near *T*_SR_ (see Ref. [2]). The diminished magnetic anisotropy near *T*_SR_ stabilizes non-coplanar spin configurations under an applied field, accounting for both incomplete magnetization saturation and the concomitant topological Hall response. At ~ 1.7 T (Fig. S3e), magnetization approaches full saturation, and the topological Hall signal accordingly vanishes.

# 3. Anisotropic magnetoresistance effect in Fe_3_Ge

In general, the peak-like feature observed in the MR curves of magnetic materials can be attributed to the anisotropic magnetoresistance (AMR) effect ^5^, which implies that the resistivity is higher when the magnetization is parallel ($\rho_{\parallel}$) to the applied current than when they are perpendicular ($\rho_{\perp}$), i.e., $\rho_{\parallel}>\rho_{\perp}$. Specifically, when an external magnetic field is applied along the hard axis (the *c*-axis for Fe_3_Ge below *T_SR_*), the spins are tilted from the easy *ab*-plane. This tilting alters the relative orientation between the current and the magnetization from perpendicular in the absence of field to parallel beyond the saturation field ${\mu_{0}H}_{s}$. This reorientation leads to an increase in resistivity, manifesting a positive MR, i.e., the peak-like feature, and therefore the magnetic field ${\mu_{0}H}_{p}$ corresponding to the MR peak should be identical to the ${\mu_{0}H}_{s}$. As shown in Fig. S4, the peak position ${\mu_{0}H}_{p}$in the MR curves matches well with ${\mu_{0}H}_{s}$ extracted from the *M-H* curves. It is worth noting that, in order to avoid complications arising from different demagnetizing factors, both the electronic transport and magnetization measurements were performed on samples with identical macroscopic geometry and comparable dimensions. These results demonstrate that the peak-like feature in the longitudinal MR of Fe_3_Ge indeed originates from the AMR effect.

**Figure S4.** Longitudinal MR with *μ*_0_*H* ∥ *I* ∥ *z* and the *M*-*H* curves with *μ*_0_*H* ∥ *z* at various temperatures.

# 4. Band structure of Fe_3_Ge in three distinct ferromagnetic phases

Fig. S5 presents the analysis of band structure and Fermi surface properties in Fe_3_Ge in three distinct ferromagnetic (FM) phases. The schematic in Fig. S3a illustrates the three magnetic configurations where Fe moments are oriented along different crystallographic directions. Below *E_F_*, two sets of Dirac points are identified (Fig. S5b-d). It is noted that, the electronic band structure calculations reveal distinct magnetic anisotropy effects on the electronic properties. For Fe moments aligned along the *x*- and *y*-axes, the band structures are remarkably similar, indicating comparable electronic environments for these in-plane magnetization directions. In contrast, when the Fe moments are oriented along the *z*- axes, significant differences emerge: the two Dirac fermions respond quite differently, with the Dirac gap at point 1 being significantly enhanced, while that at point 2 is only weakly affected. Meanwhile, the bands around the Fermi level along the M-K path become notably flattened. In addition, along the Γ-A-L-H-A path, the bands transition from a degenerate state to a split configuration, reflecting the lifting of degeneracy due to the out-of-plane magnetization. These band structure modifications highlight the strong magnetocrystalline anisotropy in Fe_3_Ge, where the *z*-direction magnetization significantly alters the electronic structure compared to the in-plane orientations. The four bands crossing the Fermi level, emphasized in different colors, indicate the metallic nature of Fe_3_Ge and the multi-band character contributing to its transport properties. The corresponding Fermi surface morphologies (Fig. S5e-g) in the *k_z_* = 0 plane reveal three closed Fermi surfaces centered around the Γ point, with the outermost surface exhibiting clear six-fold symmetry reflecting the hexagonal crystal structure, while the two inner surfaces show nearly circular shapes.

**Figure S5.** **a** Schematic illustration of three ferromagnetic phases in Fe_3_Ge with the Fe magnetic moments aligned along the *x*-, *y*-, and *z*-axes. The coordinate system is defined such that the *x*- and *z*-axes align with the crystallographic *a*- and *c*-axes, respectively, while the *y*-axis lies in the *ab* plane at 30° to the *b*-axis. **b-d** Electronic band structures of Fe_3_Ge for the three ferromagnetic phases, corresponding to Fe magnetic moments of *M_x_* ~ 2.183 *µ_B_*/Fe, *M_y_* ~ 2.232 *µ_B_*/Fe, and *M_z_* ~ 2.178 *µ_B_*/Fe, respectively. Four bands crossing the Fermi level are highlighted in different colors. Two sets of Dirac points below *E_F_* are highlighted by the blue and red circles. **e-g** Fermi surfaces of Fe_3_Ge in the *k_z_* = 0 plane for the respective ferromagnetic phases.

Moreover, we calculated the intrinsic anomalous Hall conductivity $\sigma_{zx}^{A}$ for different spin configurations, as shown in Fig. S6. It is found that when the Fe moments are aligned along the *y*-axis, the AHC is larger than when the Fe moments are oriented along the *z*-axis, which is consistent with previous reports ^6^. These results indicate that the spin configuration has a significant impact on the transport properties of Fe_3_Ge.

**Figure S6.** Intrinsic anomalous Hall conductivity $\sigma_{zx}^{A}$, plotted as a function of binding energy, calculated for different spin configurations.

# 5. Easy-plane and easy-axis magnetic anisotropy in Fe_3_Ge

**Figure S7.** Normalized *M*-*H* curves with *μ*_0_*H* ∥ *x* and *μ*_0_*H* ∥ *y* at 300 K and 100 K.

To examine in-plane magnetic anisotropy, we measured *M*-*H* curves with the magnetic field applied along the *x* ([$2\bar{1}\bar{1}0$])) and *y* ([$01\bar{1}0$]) directions, as shown in Fig. S7. The results reveal no discernible difference between the two IP directions, indicating nearly isotropic in-plane magnetic property. Similar in-plane magnetic isotropy has also been reported in other distorted kagome magnets, such as Mn_3_Sn ^7^.

**Figure S8.** *M*-*H* curves of Fe_3_Ge measured under in-plane (IP) and out-of-plane (OOP) magnetic field configurations at various temperatures.

As shown in Fig. S8, the magnetic anisotropy of Fe_3_Ge exhibits a clear temperature-dependent evolution. When the temperature is above *T*_SR_, OOP *M*-*H* curves consistently display lower ${\mu_{0}H}_{s}$, indicating that the *c*-axis acts as the magnetic easy axis. This behavior is characteristic of easy-axis magnetic anisotropy (EAMA). In contrast, upon cooling below *T*_SR_, the IP *M*-*H* curves exhibit lower ${\mu_{0}H}_{s}$, suggesting that the easy axis switches into the *ab*-plane. This change is a hallmark of easy-plane magnetic anisotropy (EPMA). These results indicate that Fe_3_Ge undergoes a spin reorientation transition at *T*_SR_, accompanied by a change in magnetic anisotropy from EAMA to EPMA.

# 6. Lorentz transmission electron microscopy in Fe_3_Ge

The LTEM experiments were performed on thin lamellae, whose geometry indeed leads to substantially different demagnetization conditions compared to bulk samples. We therefore do not use the LTEM data for quantitative comparisons of critical magnetic fields with bulk magnetization or transport results. Instead, LTEM is employed to directly visualize the evolution of magnetic textures. Importantly, despite the altered demagnetization conditions in the lamella geometry, skyrmions are observed only above the spin reorientation temperature. This behavior is fully consistent with the easy-axis magnetic anisotropy independently established by bulk magnetization measurements, indicating that the key conclusions regarding the magnetic anisotropy crossover and the stabilization of skyrmions are robust against sample geometry effects.

**Figure S9.** LTEM images (top row) and the corresponding magnetic induction maps (bottom row) of a Fe_3_Ge lamella recorded under zero magnetic field at various temperatures. For *T* < *T_SR_*, in-plane domains are observed, and magnetic induction maps reveal the presence of spin vortices and antivortices located at domain boundaries. As the temperature increases (*T_SR_* < *T* < 650 K), the labyrinthine domains emerge as the ground states at zero magnetic field. Upon further heating to 650 K, the magnetic domain contrast disappears, indicating the Curie temperature (*T*_C_) of Fe_3_Ge.

**F****igure S10.** Over-focused LTEM images of spin textures in Fe_3_Ge at 420 K under various magnetic fields.

**Figure S11.** Schematic energetics of a skyrmionic system with helicity polarization, where a small DMI contribution lowers the formation energy of skyrmions with *γ* = π/2 ^8,9^.

**Figure S12.** Over-focused LTEM images of spin textures in Fe_3_Ge during heating under zero magnetic field.

**Figure S13.** Over-focused LTEM images of spin textures in Fe_3_Ge during heating under 700 mT.

# 7. Mechanisms of skyrmion formation

Various mechanisms can generate skyrmions in magnetic systems, including Dzyaloshinskii-Moriya interaction (DMI), long-ranged magnetic dipolar interactions, frustrated exchange interactions, and four-spin exchange interactions (Table S3) ^10^. Since magnetic skyrmions were primarily discovered in noncentrosymmetric lattices or multilayer thin films, the early investigators grappled with notions that DMI is the main driver of the skyrmion formation as the DMI only appear when the crystal field has a broken inversion symmetry with respect to the center between two magnetic ions. This creed was later challenged by the observation of skyrmion lattice in centrosymmetric magnets with frustrated exchange interactions, as in the kagome or triangular-lattice magnets, where the DMI is absent. In addition, the other mechanism, namely the long-range magnetic dipolar interaction, may also contribute to the skyrmion nucleation in some magnets. In such cases, the competing perpendicular easy-axis magnetocrystalline anisotropy and magnetic dipolar interactions that usually favor the in-plane alignment of the moments, coexist and rival, resulting in the frustrated magnetic ground state and ultimately the periodic array of magnetic skyrmions. Alternative mechanism also involves the biquadratic exchange and four-spin exchange interactions, which can lead to atomic-sized skyrmion structures.

The size of skyrmions is different for these distinct origins. The size of skyrmions caused by DMI is typically in the range of 5-100 nm. For the skyrmion lattice in centrosymmetric magnets with frustrated exchange interactions, the size is of an order of the lattice constant, i.e., ~ 1 nm. The similar size of skyrmions holds for four-spin exchange interactions. In contrast, skyrmions induced by magnetic dipolar interactions have a relatively large size, i.e., of an order of 100 nm to 1 µm, significantly larger than the lattice constants. Consequently, skyrmions stabilized by DMI or dipolar interactions are larger than the lattice constant, justifying the use of a continuum approximation. In both cases, the skyrmion energy density is much smaller than the atomic exchange energy, ensuring topological protection. These large-scale skyrmions possess multiple internal degrees of freedom, rendering them highly mobile and largely free from commensurability pinning by the crystal lattice.

**Table S3.** Mechanisms of skyrmion formation^10^.

|  | The typical size of skyrmions |
| --- | --- |
| Dzyaloshinskii-Moriya interaction | 5-100 nm |
| Long-ranged magnetic dipolar interactions | 100 nm-1µm |
| Frustrated exchange interactions | ~ 1 nm |
| Four-spin exchange interactions | ~ 1 nm |

In Fe_3_Ge, as seen in Fig. 1, it crystallizes in the hexagonal D0_19_ type structure with the space group *P*6_3_/*mmc*. This hexagonal D0_19_ structure has a global inversion center and can thus be regarded as centrosymmetric in general. In this vein, the DMI is vanishingly small and cannot be the source of the skyrmion formation in Fe_3_Ge. Nevertheless, the lattice of Fe_3_Ge displays an in-plane distortion known as a breathing kagome, generating triangles with two different sizes in the kagome plane, as highlighted in Fig. 1c of the main text. This marginal distortion may generate a small DMI component such that the otherwise *c*-oriented moments are tilted towards the *ab* plane by a small angle, leading to the formation of skyrmions. As for Fe_3_Ge, the small distortion of kagome lattices may also lead to a local DM tensor. However, the size of skyrmion visualized by LTEM is about 250 nm, which is much larger than typical DMI- and frustrated-exchange-interactions-driven skyrmions, conforming to that induced by magnetic dipolar interactions. Moreover, the coexistence of opposite helicities is the hallmark of dipolar skyrmions. Dipolar skyrmions with a helicity of −π/2 and π/2 are degenerate in energy and thus should be equally populated, as illustrated in Fig. S11. In Fe_3_Ge, the observation of equally populated skyrmions with opposite helicities (−π/2 and π/2) aligns with a dipolar origin; proper modulation of the EAMA and dipolar interactions enables the stabilization of dipolar skyrmions. Typically, these dipolar skyrmions evolve from labyrinth domains within a specific range of magnetic fields, as illustrated in Fig. 4 of the main text. Collectively, these results strongly demonstrate that Fe_3_Ge can host dipolar skyrmions and trivial bubbles concurrently.

# 8. Comparison of centrosymmetric ferromagnets

**Table S4.** A summary of some key parameters of centrosymmetric topological ferromagnets ^11-23^. Skyrmion temperature windows are based on in-situ Lorentz transmission electron microscopy observations.

|  | *T*_C_ (K) | *T*_sk_ (K) | *d*_sk_ (nm) | Stabilization mechanism | Semimetal category | AHC (Ω^-1^cm^-1^) |
| --- | --- | --- | --- | --- | --- | --- |
| Fe_3_Ge | 650 | 375-650 | ~ 250 | Dipolar interaction | Dirac | ~380 (300 K) |
| Mn_5_Ge_3_ | 285 | 100-260 | ~110 | Dipolar interaction | Weyl | ~190 (250 K) |
| Fe_3_Sn_2_ | 640 | 100-640 | ~ 280 | Dipolar interaction | Dirac | ~125 (300 K) |
| Fe_3_GeTe_2_ | 220 | 100-220 | ~ 100 | Defect driven DMI | Nodal lines | ~150 (210K) |
| Fe_5_GeTe_2_ | 275 | 100-270 | ~ 120 | Dipolar interaction | Nodal lines | ~ 70 (250K) |
| Fe_3_GaTe_2_ | 350 | 100-350 | ~ 100 | Defect driven DMI | Nodal lines | ~ 220 (300 K) |
| TbMn_6_Sn_6_ | 423 | 310-400 | ~ 100 | RKKY interaction | Dirac | ~180 (300 K) |

Table S4 summarizes several representative centrosymmetric topological ferromagnets ^11-23^, and compares key parameters such as the Curie temperature (*T*_C_), anomalous Hall conductivity (AHC), skyrmion stability temperature window (*T*_sk_), typical skyrmion diameter (*d*_sk_), and the dominant stabilization mechanism. This comparison highlights that Fe_3_Ge uniquely combines a high Curie temperature, a large anomalous Hall response, and an exceptionally wide high-temperature skyrmion stability window among these ferromagnets.

# 9. Magneto-optical Kerr effect measurement setup

To clarify the measurement geometry, Fig. S14 presents a schematic illustration of the magneto-optical Kerr effect setup, indicating the directions of the magnetic field, light incidence, and crystallographic axes.

**Figure S14.** Schematic illustration of the magneto-optical Kerr effect measurement setup.

**10. Geometric tunability of mixed textures in Fe_3_Ge**

The stability and characteristic size of dipolar skyrmions are governed primarily by the balance between dipolar interaction, magnetic anisotropy, exchange interaction, and demagnetization energy. Unlike DMI-stabilized skyrmions, dipolar skyrmions do not rely on a fixed chiral energy scale, and therefore exhibit strong sensitivity to extrinsic geometric parameters such as sample thickness and lateral confinement.

In thin magnetic systems, previous experimental and theoretical studies have demonstrated that reducing the sample thickness effectively modifies the demagnetizing field distribution and enhances shape anisotropy. This leads to a reduction in the characteristic domain size and stabilizes smaller skyrmionic textures. Concurrently, geometric confinement (e.g., patterning into nanostructures such as strips, dots, or constrictions) further imposes boundary conditions that restrict domain wall expansion and suppress large-scale labyrinthine domains. As a result, trivial bubbles, which are typically stabilized by larger-scale domain competition, become energetically unfavorable under confined geometries. Consequently, both the skyrmion size and inter-skyrmion spacing can be continuously tuned by engineering sample geometry. In addition, confinement-induced modification of the magnetostatic energy landscape can selectively stabilize skyrmionic textures while eliminating non-topological bubble states.

In the context of Fe_3_Ge, these effects suggest that the coexistence of skyrmions and trivial bubbles observed in bulk-like lamellae reflects an intermediate thickness regime where dipolar, anisotropy, and exchange energies are comparable. We therefore expect that further reducing the thickness or introducing lithographically defined confinement structures would (i) reduce the skyrmion size, (ii) enhance skyrmion uniformity, and (iii) suppress trivial bubble formation, thereby enabling a more controllable and denser skyrmion ensemble.

These tunability features provide a practical pathway toward functional device architectures based on dipolar skyrmions, particularly for high-density information storage and spintronic logic applications where controllable nanoscale spin textures are required.

# 11. Videos of skyrmion formation

**Movies S1** Spin-texture evolution in Fe_3_Ge under 0.72 T upon heating from 300 K to 650 K.

**References**

1	Li, S.-X. *et al.* Giant Anomalous Hall Effect in Kagome Nodal Surface Semimetal Fe_3_Ge. *arXiv preprint arXiv:2501.15424* (2025). <https://doi.org/doi.org/10.48550/arXiv.2501.15424>

2	Xu, C. *et al.* Large Anomalous and Topological Hall Effect and Nernst Effect in a Dirac Kagome Magnet Fe_3_Ge. *Advanced Functional Materials* **n/a**, e11059 (2025). <https://doi.org/doi/10.1002/adfm.202511059>

3	Li, Z. *et al.* Anomalous Hall effect dominated by intrinsic mechanism in Fe_3_Ge with hexagonal DO_19_ Kagome lattice and cubic DO_3_ structure. *Applied Physics Letters* **122** (2023). <https://doi.org/10.1063/5.0118768>

4	Zhang, Z. *et al.* Large topological Hall effect arising from spin reorientation in kagome magnet Fe_3_Ge. *arXiv preprint arXiv:2403.17354* (2024). <https://doi.org/doi.org/10.48550/arXiv.2403.17354>

5	Ritzinger, P. & Výborný, K. Anisotropic magnetoresistance: materials, models and applications. *Royal Society Open Science* **10**, 230564 (2023). <https://doi.org/doi:10.1098/rsos.230564>

6	Ghosh, S., Bhowmik, T. K., Low, A. & Thirupathaiah, S. Spin-reorientation-driven temperature-dependent intrinsic anomalous Hall conductivity observed in Fe_3_Ge: A ferromagnetic topological metal. *Physical Review Materials* **9**, 084201 (2025). <https://doi.org/10.1103/lsjt-cww1>

7	Nakatsuji, S., Kiyohara, N. & Higo, T. Large anomalous Hall effect in a non-collinear antiferromagnet at room temperature. *Nature* **527**, 212-215 (2015). <https://doi.org/10.1038/nature15723>

8	Göbel, B., Mertig, I. & Tretiakov, O. A. Beyond skyrmions: Review and perspectives of alternative magnetic quasiparticles. *Physics Reports* **895**, 1-28 (2021). <https://doi.org/https://doi.org/10.1016/j.physrep.2020.10.001>

9	Zhang, C. *et al.* Magnetic Skyrmions with Unconventional Helicity Polarization in a Van Der Waals Ferromagnet. *Advanced Materials* **34**, 2204163 (2022). <https://doi.org/https://doi.org/10.1002/adma.202204163>

10	Nagaosa, N. & Tokura, Y. Topological properties and dynamics of magnetic skyrmions. *Nature Nanotechnology* **8**, 899-911 (2013). <https://doi.org/10.1038/nnano.2013.243>

11	Li, H. *et al.* Emergent Magnetic Skyrmions in a Topological Weyl Nodal Ring Semimetal. *Nano Letters* **25**, 2903-2910 (2025). <https://doi.org/10.1021/acs.nanolett.4c06259>

12	Low, A., Bhowmik, T. K., Ghosh, S., Changdar, S. & Thirupathaiah, S. Topological Hall effect in ferromagnetic Weyl semimetal ${\mathrm{Mn}}_{5}{\mathrm{Ge}}_{3}$ originating in competing dipolar interaction and magnetocrystalline anisotropy. *Physical Review B* **111**, 144422 (2025). <https://doi.org/10.1103/PhysRevB.111.144422>

13	Hou, Z. *et al.* Observation of Various and Spontaneous Magnetic Skyrmionic Bubbles at Room Temperature in a Frustrated Kagome Magnet with Uniaxial Magnetic Anisotropy. *Advanced Materials* **29**, 1701144 (2017). <https://doi.org/https://doi.org/10.1002/adma.201701144>

14	Ye, L. *et al.* Massive Dirac fermions in a ferromagnetic kagome metal. *Nature* **555**, 638-642 (2018). <https://doi.org/10.1038/nature25987>

15	Wang, Q., Sun, S., Zhang, X., Pang, F. & Lei, H. Anomalous Hall effect in a ferromagnetic Fe_3_Sn_2_ single crystal with a geometrically frustrated Fe bilayer kagome lattice. *Physical Review B* **94**, 075135 (2016). <https://doi.org/10.1103/PhysRevB.94.075135>

16	Chakraborty, A. *et al.* Magnetic Skyrmions in a Thickness Tunable 2D Ferromagnet from a Defect Driven Dzyaloshinskii–Moriya Interaction. *Advanced Materials* **34**, 2108637 (2022). <https://doi.org/https://doi.org/10.1002/adma.202108637>

17	Kim, K. *et al.* Large anomalous Hall current induced by topological nodal lines in a ferromagnetic van der Waals semimetal. *Nature Materials* **17**, 794-799 (2018). <https://doi.org/10.1038/s41563-018-0132-3>

18	Lv, X. *et al.* Manipulating the Magnetic Bubbles and Topological Hall Effect in 2D Magnet Fe5GeTe2. *Advanced Functional Materials* **34**, 2308560 (2024). <https://doi.org/https://doi.org/10.1002/adfm.202308560>

19	May, A. F., Bridges, C. A. & McGuire, M. A. Physical properties and thermal stability of Fe_5-_*_x_*GeTe_2_ single crystals. *Physical Review Materials* **3**, 104401 (2019). <https://doi.org/10.1103/PhysRevMaterials.3.104401>

20	Li, Z. *et al.* Room-temperature sub-100 nm Néel-type skyrmions in non-stoichiometric van der Waals ferromagnet Fe_3-_*_x_*GaTe_2_ with ultrafast laser writability. *Nature Communications* **15**, 1017 (2024). <https://doi.org/10.1038/s41467-024-45310-2>

21	Cho, W. *et al.* Singular Hall Response from a Correlated Ferromagnetic Flat Nodal-Line Semimetal. *Advanced Materials* **36**, 2402040 (2024). <https://doi.org/https://doi.org/10.1002/adma.202402040>

22	Zhang, H. *et al.* Exchange-biased topological transverse thermoelectric effects in a Kagome ferrimagnet. *Nature Communications* **13**, 1091 (2022). <https://doi.org/10.1038/s41467-022-28733-7>

23	Li, Z. *et al.* Discovery of Topological Magnetic Textures near Room Temperature in Quantum Magnet TbMn6Sn6. *Advanced Materials* **35**, 2211164 (2023). <https://doi.org/https://doi.org/10.1002/adma.202211164>
